# Alpha7 Nicotinic Acetylcholine Receptor Is a Target in Pharmacology and Toxicology

**DOI:** 10.3390/ijms13022219

**Published:** 2012-02-17

**Authors:** Miroslav Pohanka

**Affiliations:** Faculty of Military Health Sciences, University of Defence, Trebesska 1575, 50001 Hradec Kralove, Czech Republic; E-Mail: miroslav.pohanka@gmail.com; Tel.: +420-973253091; Fax: +420-973253091

**Keywords:** inflammation, cholinergic anti-inflammatory pathway, schizophrenia, Alzheimer’s disease, cognitive disorder, agonist, antagonist

## Abstract

Alpha7 nicotinic acetylcholine receptor (α7 nAChR) is an important part of the cholinergic nerve system in the brain. Moreover, it is associated with a cholinergic anti-inflammatory pathway in the termination of the parasympathetic nervous system. Antagonists of α7 nAChR are a wide group represented by conotoxin and bungarotoxin. Even Alzheimer’s disease drug memantine acting as an antagonist in its side pathway belongs in this group. Agonists of α7 nAChR are suitable for treatment of multiple cognitive dysfunctions such as Alzheimer’s disease or schizophrenia. Inflammation or even sepsis can be ameliorated by the agonistic acting compounds. Preparations RG3487, SEN34625/WYE-103914, SEN12333, ABT-107, Clozapine, GTS-21, CNI-1493, and AR-R17779 are representative examples of the novel compounds with affinity toward the α7 nAChR. Pharmacological, toxicological, and medicinal significance of α7 nAChR are discussed throughout this paper.

## 1. Introduction

Pharmacological neuromodulation has become one of the suitable tools for influencing the whole system of the human body. However, the interest in this is undermined by the fact that drugs specifically implicated in neuromodulation will be more potent than the ones influencing the periphery due to their effect amplification. Nevertheless, the current attention is aimed at the treatment of illnesses associated with neuropathology e.g., schizophrenia, Alzheimer’s and Parkinson disease.

Because the nerve system is connected with many processes, the neuromodulation can be used for alteration of, e.g., the immune system or to slow down degenerating processes. This review is focused on α7 nicotinic acetylcholine receptor as a pharmacological target. The receptor is well known for its role in the central nervous system but only recently a connection of the immune system and parasympathetic nervous system was recognized. Future perspectives are introduced here.

## 2. Acetylcholine as Neurotransmitter

The cholinergic system is one of the excitatory pathways participating in the parasympathicus, sympathicus, and the central nervous system using acetylcholine as a neurotransmitter [1]. Loewi was the first investigator that proposed the physiological importance of the simple compound acetylcholine in the 1920s [2]. Acetylcholine and acetylcholine receptors are known to be present on many cell types including endothelial cells and cells of the immune system [3].

Acetylcholine is synthesized from two precursors: acetyl coenzyme A and choline using enzyme choline O-acetyltransferase (ChAT; EC 2.3.1.6). ChAT with higher activity is localized in cytosol near neurosynapses as a soluble molecule. However, ChAT can be also found as a membrane bound structure [4]. The localization of ChAT in neurons by immunohistopathology is a basic tool for distinguishing between cholinergic and adrenergic nerve system [5]. Acetylcholine established in neurons is antiported by protons into vesicles using vesicular acetylcholine transporter (VAChT). Interaction of vesicles with the neurosynapse membrane is possible only if change in calcium level inside the cell takes place. The process is assisted by vesicle-associated membrane proteins (VAMP). The absorbed calcium molecules interact with C2A and C2B subunits of synaptotagmin [6]. Synaptoprevin in the presence of calcium forms a complex with synaptosomal-associated protein 25 (SNAP-25) which is weakly bound on the inner membrane of synapse [7]. Subsequently, syntaxin, another molecule on the inner membrane undergoes interaction with the complex resulting in the final formation of a complex soluble N-ethylmaleimide-sensitive fusion factor attachment protein receptors (SNARE) necessary for acetylcholine vesicles fusion with membrane [8]. Acetylcholine is released by fusion of the vesicles and cell membranes and interacts with acetylcholine receptors (AChR).

**Figure 1:**
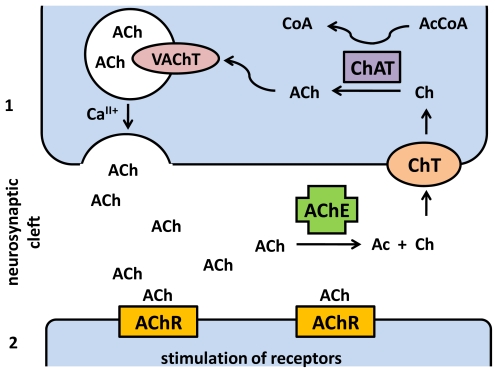
Overview of cholinergic neurotransmission: ACh—acetylcholine; Ac—acetate; AChE—acetylcholinesterase; AChR—acetylcholine receptor; ChAT—choline O-acetyltransferase; ChT—high-affinity choline transporter; VAChT—vesicular acetylcholine transporter, 1—axonal termination of neuron; 2—dendrite of neuron or other target cell.

Acetylcholine is a chemically stable compound that can persist for a long time after spreading into the neurosynaptic cleft and spontaneous elimination is slow due to the quaternary ammonium atom in the choline moiety. For this reason, enzyme acetylcholinesterase (AChE) is present in the neurosynaptic cleft to quickly terminate the signal. AChE splits acetylcholine into acetic acid and choline [9] while choline is transported from the neurosynaptic cleft using high-affinity choline transporter (ChT) back to the cytosol, acetic acid is further decomposed [10]. The complete scheme of cholinergic neurotransmission is depicted in Figure 1.

In addition to AChRs and AChE, other proteins related to vesicles fusion are also targets of numerous toxins e.g., butulotoxins. These endopeptidases are produced by bacteria *Clostridium botulinum* and less commonly by *C. baratii* and *C. butyricum* [11,12]. Butulotoxin is a dimer composed of 100 kDa heavy chain and 50 kDa light chain with Zn^II+^ endopeptidase activity [13]. There are seven basic isoforms of butulotoxine (A, B, C, D, E, F, and G) with different substrate selectivity [14]. A, C and E split SNAP 25 while B, D, F and G split synaptobrevin. The last type C splits syntaxin.

## 3. Acetylcholine Receptors

Two types of AChRs are known: muscarinic (mAChR) and nicotinic (nAChR). Names are derived from responds to interactions with secondary metabolites recognized as selective agonists. Muscarine is a fungal natural parasympathomimetic from the fly amanita (*Amanita muscaria*). The mAChRs can be found in the central as well as peripheral nervous system. In an extensive level it is inherent in the neuromuscular junction and endocrine glands. In comparison with nAChR, mAChR are coupled with G proteins [15]. In compliance with the former approach, mAChRs were divided into two separate groups: the first was stimulatory, connected with the activity of phospholipase C, the second type is inhibitory based on the suppression of adenylate cyclase activity [16]. Five types of mAChRs are currently known: M1, M2, M3, M4 and M5 [17]. The types M1, M3, and M5 are connected with the accumulation of intracellular calcium and activation of phospholipases. The preference junction of the M1, M3 and M5 is toward G_q_ and G_11_ [18,19]. The last subtypes M2 and M4 inhibit adenylate cyclase via G_i_ or G_z_ [20,21]. Atropine is a known secondary metabolite from solanum plants (*Solanaceae*) and is probably the best-known antagonist of acetylcholine on mAChR. Atropine is widely used for the treatment of bradycardia and asystole [22] and can also protect mAChR from overstimulation when AChE becomes inhibited e.g., by nerve agents [23]. Scopolamine is another antagonst of M1 receptors used in ophthalmology and for suppression of nausea [24]. Modulation of mAChR can be also suitable for the treatment of psychotic disorders [25]. Stimulation of M1 and M4 can suppress effect of amphetamines [26] and stimulation of M1 is perspective in the development of drugs for schizophrenia treatment [27]. Combination of M1 agonists and AChE inhibitors promotes REM sleep [28].

Nicotinic acetylcholine receptors (nAChR) are named after natural agonist nicotine, a secondary metabolite from a common tobacco plant (*Nicotiana tabacum, Solanaceae*). Nicotine alone is used to wean patients from smoking and in combination with other ingredients of cigarette smoke reduces schizophrenia manifestations [29,30]. However, it is unclear whether nicotine influences cognitive functions during schizophrenia by nAChR agonism or if it is implicated in monoamine oxidase activity modulation [31]. Currently, clinical trials are conducted to confirm nicotine’s benefits to patients with Parkinson’s disease, schizphrenia, sarcoidosis, and as pain relief [32]. It is widely accepted that nAChRs are present in both central and peripheral sections of the nerve system and neuromuscular junctions. In addition, evidence suggests nicotine also participates in the nerve-immune system junction [33] as introduced later.

In mammals, analysis of cDNA proved existence of 17 homologues of nAChR’s subunits divided into five subtypes: α1-10, β1-4, γ, δ, ɛ [34]. Except of the avian subtype α8, all subtypes can be found in mammals [35]. One nAChR molecule is composed from five subunits formed in radial symmetry with central pore. Each subunit includes four transmembrane α helixes, large intracellular domain composed from one α helix and extracellular part with affinity to acetylcholine and other agonists [36]. Acetylcholine is bound in a cavity formed by the disulfide bridge from the two cysteine residues. From the known subtypes, the cavity is formed by α1, α2, α3, α4, α6, α7 and α8 subtypes only. The other subtypes, α1, β2, β4, δ, γ and ɛ, may stabilize cavity from the outside [37]. Homopentamer α7 as well as some other receptors have the affinity not only to acetylcholine but also to choline [38]. The individual subtypes can be combined one to each other with different stechiometry. The (α7)_5_, (α4)_2_(β2)_3_, (α4)_3_(β2)_2_ a (α4)_2_(β2)_2_α5 nAChR are the most common [39,40]. Mechanism of the nAChR’s function is the same for all receptors regardless to presence of different subtypes. It forms an ion channel every time. However, the main difference is in which ions are allowed to flow through the receptor. The presence of polar regions and/or subtype α3 will allow selective flow of Na^I+^ and K^I+^. On the other hand, non-polar residua and a high level of glutamic acid typical for the α7 nAChR homopentamer, enhance specificity to Ca^II+^ [41].

nAChRs are influenced by several toxins and pharmacologically applicable compounds e.g., α bungarotoxin from the Taiwan krait *Bungarus multicinctus*. It is a strong antagonist of nAChRs with partial selectivity to α7 nAChR [42]. nAChRs are considered to be a strong antagonist on succinylcholine (suxamethonium), they are used for recovery from near fatal hyperthermia [43] and neuromuscular junction paralysis in anesthesia [44]. Since succinylcholine is converted by plasmatic butyrylcholinesterase, butyrylcholinesterase dysfunctional patients are extremely sensitive to succinylcholine and recovery from its application lasts longer when compared to individuals with fully active butyrylcholinesterase [45].

## 4. α7 Nicotinic Acetylcholine Receptor in Brain

The α7 nAChR is implicated in cognitive functions of the central nervous system. Modulation of α7 nAChR is considered to be perspective for the treatment of cognitive disorders such as Alzheimer’s disease or schizophrenia. Agonists of α7 nAChR are able to penetrate through the blood brain barrier. They are objects of pharmacologists’ interest as they are suitable for cognitive function amelioration in patients suffering from Alzheimer’s disease and schizophrenia [46]. Moreover, amyloid β released in Alzheimer’s disease patients also extensively binds to the brain α7 nAChR and prevents its natural function [47]. As acetylcholine level is limited in Alzheimer’s disease damaged brain agonizing α7 nAChR is considered as a promising way to enhance cognitive functions [48]. Feher *et al.* investigated the link between α7 nAChR and some types of dementia [49]. They recognized significantly elevated 2 bp deletion in α7 nAChR subunit gene in individuals suffering from Alzheimer’s disease, dementia with Lewy bodies, and Pick’s disease. Moreover, actual α7 nAChR is over-expressed in patients with Alzheimer’s disease [50]. These findings should be extensively researched and molecular mechanism of α7 nAChR in neurodementias has to be better understood before any conclusions can be made.

Experimental application of α7 nAChR agonists can be beneficial in schizophrenia treatment e.g., Tregellas *et al.* successfully tested 3-(2,4-dimethoxybenzylidene) anabaseine as a α7 nAChR agonist [51]. It is vital that cholinergic nerves can modulate release of dopamine and glutamate. Livingston *et al.* proved that α7 nAChR might be stimulated by choline. Moreover, they tested compound PNU-120596 (see later) and proved that it elicits dopamine release in the rat prefrontal cortex [52]. Effectiveness of α7 nAChRs agonists is intriguing for schizophrenia treatment. Drugs for schizophrenia Clozapine and 3-(2,4-dimethoxybenzylidene) anabaseine can be used as an example of drugs implicated in α7 nAChR agonism [53]. Special animal models have been introduced in order to investigate the α7 nAChR modulation by chemical compounds for, e.g., Alzeheimer’s disease and other disorders [54–56].

## 5. Cholinergic Anti-Inflammatory Pathway

**Figure 2:**
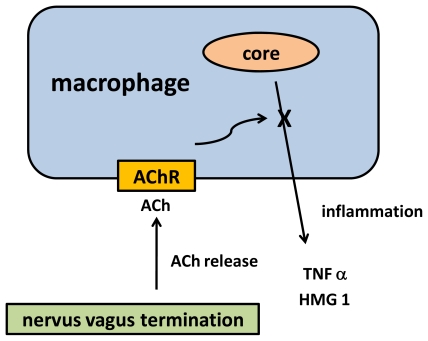
Scheme of cholinergic anti-inflammatory pathway: ACh—acetylcholine; TNF α—tumor necrosis factor α; HMG 1—high-mobility group protein 1.

Cholinergic anti-inflammatory pathway is a link between parasympathetic and innate immune system. Tracey and co-workers firstly described it as they recognized nervus vagus in immunomodulation and called it inflammatory reflex [57]. Scheme of cholinergic anti-inflammatory pathway is depicted as Figure 2. Macrophages are able to produce pro-inflammatory cytokines e.g., tumor necrosis factor α (TNFα) and expression of high-mobility group protein 1 (HMG 1) with intracellular as well as extracellular signalization function. Regarding cholinergic anti-inflammatory pathway, principal parasympathetic terminations in the blood system are able to release acetylcholine that interacts with α7 nAChR on macrophages surface. Macrophage-assisted inflammation is stopped after the receptor stimulation. During inflammation the α7 nAChR action is associated with calcium influx and stop of nuclear factor κB (NF κB) stimulation [58–60].

Inflammatory processes can be deteriorating without being properly controlled. Septic shock can be mentioned as an example. It is a life-threatening event with high expected mortality rate [61]. Another deteriorating action of immune system represented by macrophages is atherosclerosis [62]. Macrophages are also a target in HIV pathology as virus can proliferate within and the pathology is also involved in macrophage mediated bystander T lymphocytes apoptosis [63]. Stimulation of the cholinergic anti-inflammatory pathway is believed to be a neuro-immunomodulatory action with fast and reliable calming of the innate immune system [64]. The cholinergic anti-inflammatory pathway was proven to be effective in sepsis treatment [65], in ischemia (myocardial ischemia reperfusion injury) [66], and rheumatoid arthritis [67].

## 6. Antagonists of α7 nAChR

Agonists and antagonists of α7 nAChR are a wide group of heterogeneous compounds. Antagonists of α7 nAChR have lower practical impact in comparison with agonists. Several natural toxins can be used as examples of compounds antagonizing acetylcholine on α7 nAChR [32]. Moreover, some drugs are potent to antagonize α7 nAChR as a side effect of their main pharmacological effect. Two groups of proteins’ respective peptides are the best-known antagonists of α7 nAChR. Conotoxins are a group of cysteine-rich peptides from cone snails (*Conus* sp.) possessing various ion channel blocking. The α conotoxins selectively target the nAChR [68]. They contain two-loop frameworks and are selective to the acetylcholine binding site [69]. α connotoxins from cone snail *Conus consors* abbreviated as CnIA with sequency GRCCCHPACGKYYSC and amidated C terminus are selective and reversible antagonists of α7 nAChR [70]. However, the other α conotoxins such as α conotoxin PnIA are also antagonists of α7 nAChR with low median inhibitory concentration: 14 nM [71]. Conotoxins are potent inhibitors of nAChR when considered the median inhibitory concentration for the individual toxins. The strongest inhibitors possess median inhibitory concentration in nanomolar levels [72]. Due to their size and physical properties, conotoxins cannot simply cross the blood brain barrier. Thereby their action is preferably in the peripheral nervous system [73]. On the other hand, distribution to the central nervous system is not restricted and central action of conotoxins can be also recognized in some cases [74]. Though the conotoxins are not utilized for pharmacology purposes, they were found to be suitable for distinguishing between individual types of nAChR [75].

α Bungarotoxin is another specific antagonist of α7 nAChR. It is venom from Taiwanese krait *Bungarus multicinctus*. Beside α7 nAChR, α bungarotoxin can also bind on to GABA β3 subunit [76]. The isolation of α Bungarotoxin and understanding mechanism of action has been extensively investigated from 1960s [77,78]. α Bungarotoxin binds irreversibly to the nAChR and the complex bungarotoxin—nAChR is immunogenic, which may result in production of autoimmune antibodies. For this reason, α bungarotoxin is a suitable experimental model for myasthenia gravis [79].

**Figure 3:**
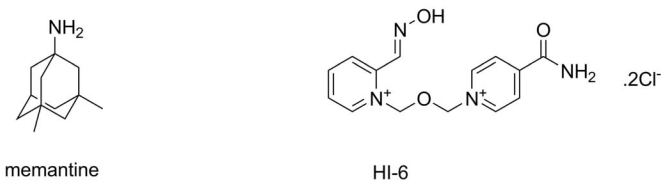
Structures of memantine and HI-6.

Memantine (3,5-Dimethyl-tricyclo[3.3.1.13,7]decan-1-amine hydrochloride) and methyllycaconitine are low molecular weight antagonists of α7 nAChR. Memantine (Figure 3) is an approved drug for treatment of Alzheimer’s disease acting as a strong NMDA receptor antagonist. Beside the action on NMDA receptors, memantine was proved to be a α7 nAChR antagonist with median inhibitory concentration 0.34 μM [80]. Although Aracava *et al.* considered antagonism of memantine on α7 nAChR as a negative phenomenon in Alzheimer’s disease treatment [80], the antagonism is considered to be beneficial in Alzheimer’s disease treatment according to other scientists [81]. However, the significance of the antagonism should be elucidated and antagonism suitability for Alzheimer’s disease treatment is not a common view; more trials are needed [82]. Methyllycaconitine is a 683 Da weighting secondary metabolite from *Consolida* genus. It is an antagonist of nAChR with the highest affinity to α7 subtype [83,84]. However, there still remains one group of nAChR antagonists that were not mentioned in the previous text: pyridinium oximes and bispyridinium oximes. These compounds are used for antidotal treatment after exposure to organophosphorous inhibitors of AChE [85]. It was proved that these compounds can bind like acetylcholine due to the quarternary ammonium atom and interact with AChE and with AChR [86,87]. Biological effects HI-6 (asoxime; Figure 3) are considered to be associated with α7 nAChR; unfortunately the plausible proof is still missing [88,89]. The antagonists of α7 nAChR are summarized in Table 1.

## 7. Agonists of α7 nAChR

α7 nAChR agonists become a group of extensively investigated compounds. As was mentioned above, agonists are perspective for the treatment of cognitive dementia, schizophrenia, inflammation and sepsis regarding to the physiological importance of α7 nAChR. However, structural requirements for these drugs are quite different. Drugs suitable for schizophrenia and dementia treatments have to be able to penetrate the blood brain barrier. On the other hand, the cholinergic anti-inflammatory pathway can be also be controlled by compounds that are not distributed into the central nervous system. The blood brain barrier penetrating compounds are typically small lipophilic molecules with no charged atom [90,91] and/or recognizable by specific carriers [92]. Piperidyl or azabicyclooctane derivatives are the most common derivatives.

**Figure 4:**
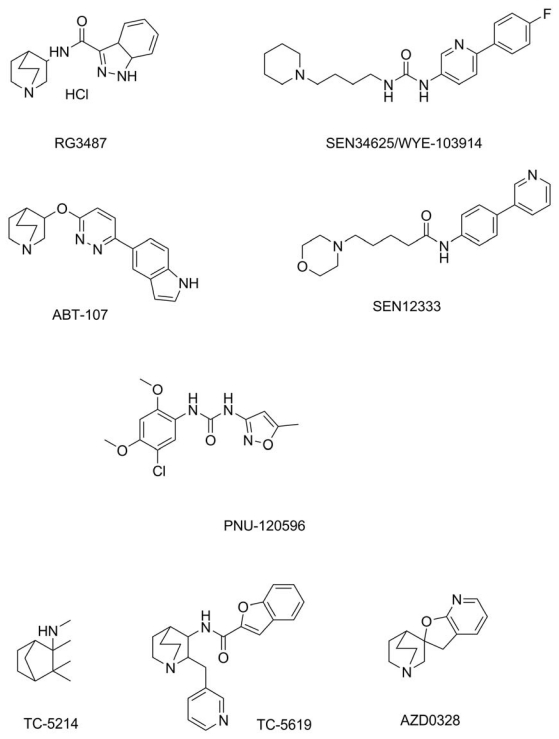
Structures of α7 nAChR agonists acting in the central nervous system.

Selected pharmacologically relevant agonists of α7 nAChR are depicted in Figure 4. Wallace *et al.* prepared compound RG3487, N-[(3S)-1-azabicyclo[2.2.2]oct-3-yl]-1H-indazole-3-carboxamide hydrochloride recognized as a human α7 nAChR agonist with high affinity represented by equilibrium constant 6 nM and median effective concentration 0.8 μM [93]. RG3487 was also proved as a potent antagonist of serotonin receptor with median inhibitory constant 2.8–33 nM. The authors proved the significant effect of the compound on rodents’ cognitive functions and discussed perspectives for Alzheimer’s disease symptomatic treatment. Moreover, it was proven to reverse phencyclidine-induced impairments in a rodent model. Another agonist of α7 nAChR for cognitive disorders treatment was introduced by Chiron *et al.* [94]. They introduced 1-[6-(4-fluorophenyl) pyridin-3-yl]-3-(4-piperidin-1- ylbutyl) urea, abbreviated as SEN34625/WYE-103914, that is a strong agonist of α7 nAChR as the median effective concentration was 70 nM. SEN34625/WYE-103914 is the more effective analogue of previously prepared compound SEN12333 (5-morpholin-4-yl-pentanoic acid (4-pyridin-3-yl-phenyl)-amide) promising for Alzheimer’s disease and schizophrenia treatment with median effective concentration to α7 nAChR 12 μM [95]. The compound crosses the blood brain barrier well and can be administered orally. ABT-107 is also an agonist of α7 nAChR structurally different to SEN compounds [96]. Median effective concentration for ABT-107 was 50–90 nM for rat oocytes. According to the author’s report, ABT-107 was also found to be effective in protecting rat cortical cultures from glutamate-induced toxicity. In the text above, clozapine (8-chloro-11-(4- methylpiperazin-1-yl)-5H-dibenzo[*b*,*e*][1,4]diazepine) and 3-(2,4-dimethoxybenzylidene) anabaseine were mentioned as drugs acting as a α7 nAChR agonists [51,53]. However, clozapine is a commercial antipsychotic for schizophrenia [97] and interaction with α7 nAChR is not the main pharmacological pathway. It is sold under different trade names e.g., Azaleptin, Clozaril, FazaClo, Leonex and others. Besides those mentioned above, some other compounds are in clinical trials and are at the center of extensive scientific interest. PNU-120596 (1-(5-chloro-2,4-dimethoxyphenyl)-3-(5-methylisoxazol-3- yl)urea) is a potent allosteric modulator [98] with good potential to act as a neuroprotective agent enhancing cognition ability [99]. Compound TC-5214 (S-(+)-mecamylamine) is nAChR antagonist with low selectivity toward individual isotypes [100]. Presently, the TC-5214 preparation is evaluated for its palliative and antidepressant effect. Promising compounds acting as a α7 nAChR selective agonists seem to be also AZD0328 and TC-5619, both containing azabicyclooctane group. They are expected to be useful to treating multiple cognitive dysfunctions and schizophrenia [101,102]. It is noteworthy that product of acetylcholine hydrolysis, choline, is an agonist of α7 nAChR, too [103].

**Figure 5:**
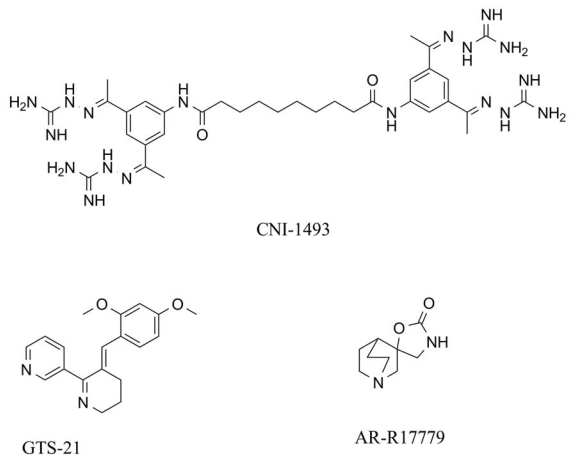
Structures of α7 nAChR agonists with significant anti-inflammatory properties.

Compounds eliciting cholinergic anti-inflammatory pathway are another group of promising drugs. Immunology principle of the cholinergic anti-inflammatory pathway was extensively reviewed recently [64,104]. Pharmacological stimulation of the pathway seems to be suitable for treatment of multiple diseases associated with over stimulation of immune system such as autoimmune disorders, sepsis, acute pancreatitis etc. [105,106]. Presently, there is no clinically approved drug primary targeted on cholinergic anti-inflammatory pathway. However, certain novel compounds seem to be promising for the pertinent clinical trials. An example is [3-(2,4-dimethoxybenzylidene) anabaseine] abbreviated as GTS-21 or DMXB-A in some sources. It is a compound found in Pacific nemertine *Paranemertes peregrina* and currently tested for treatment of Alzheimer’s disease and cognitive deficits associated with schizophrenia [107]. It is able to attenuate TNF α as well as other pro-inflammatory cytokines production. GTS-21 (Figure 5) was able to improve survival rate of BALB/c mice suffered from endotoxemia when applied in dose 4 mg/kg [108]. As proved on a C57BL6 mouse model, the compound is also suitable for prevention of inflammatory injury induced by mechanical ventilation [109]. In addition to the anti-inflammatory pathway, GTS-21 was assessed in a clinical trial and was proved to improve the cognitive functions of schizophrenic patients [110]. A tertravalent guanylhydrazone CNI-1493 (Figure 5) known also as semapimod (*N*,*N*′-bis[3,5-bis[*N*- (diaminomethylideneamino)-*C*-methylcarbonimidoyl] phenyl] decanediamide tetrahydrochloride) is an agonist of α7 nAChR primary introduced as a selective pro-inflammatory cytokine inhibitor [111] suitable for activation of cholinergic anti-inflammatory pathway [112]. In addition to this, it ameliorates amyloid β deposition and it is a perspective for treatment of cognitive deterioration in Alzheimer’s disease. It acts via suppression of inflammation with perspective to amend disease progression [113]. CNI-1493 was found to be effective for resolving of endotoxic shock in a rat model [114]. In a clinical trial, CNI-1493 was examined as a drug for the treatment of Crohn’s disease [115], a disease associated with inflammation. The investigators reported no plausible effect for CNI-1493 single and 3 day dosing. However, cumulative dosing brings some positive effects to Crohn’s disease therapy. Anti-inflammatory properties were also found for a α7 nAChR agonist AR-R17779 ((2*S*)- 2′H-spiro[4-azabicyclo[2.2.2]octane-2,5′-[1,3]oxazolidin]-2′-one) which is reported to be suitable for amelioration of ileus in mice [116]. AR-R17779 (Figure 5) can also act in the central nervous system where it can ameliorate cognitive function [117]. Selected α7 nAChR antagonists are summarized in Table 2.

## 8. Conclusions

Agonists and antagonists of α7 nAChR are pharmacologically relevant compounds suitable for treatments of multiple cognitive dysfunctions and/or inflammation associated diseases. Although drugs specifically targeted to α7 nAChR are not clinically approved, the recent investigations provided good preliminary results. In compliance with the known facts about novel compounds, increased clinical interest and clinical trials for the novel drugs can be expected.

## Figures and Tables

**Table 1:** Overview of selected α7 nAChR antagonists.

Name	Structure	Source	Use	Reference
α-bungarotoxin	8 kDa globular protein	Taiwanese krait *Bungarus multicinctus*	not used in therapy, natural toxin	[77–79]
CnIA, PnIA	polypeptide	α-conotoxins from cone snail *Conus consors*	natural toxin, use in research for distinguishing of acetylcholine receptors types	[70–75]
HI-6 (also known as asoxime)	1-[(4-carbamoylpyridin-1-ium- 1-yl)methoxymethyl]pyridin-1- ium-4- carboxamide dichloride CAS: 34433-31-3	byspyridinium oxime derivative	therapy of nerve agents poisoning via reactivation of AChE	[85–89]
memantine	3,5-Dimethyltricyclo[ 3.3.1.13,7]decan-1- amine hydrochloride CAS: 19982-08-2	adamantane derivative	Alzheimer’s disease drug antagonizing NMDA receptor, antagonism of α7 nAChR is a side pathway	[80–82]
methylcaconitine	683 Da alkaloid CAS: 21019-30-7	*Consolida* flowers	not used in therapy, natural toxin	[83–84]

**Table 2:** Overview of selected α7 nAChR antagonists.

Name	Structure	Use	Reference
ABT-107	5-(6-[(3R)-1- azabicyclo[2.2.2]oct-3- yloxy]pyridazin-3-yl)-1H-indole	Treatment of Alzheimer’s disease and cognitive deficits associated with schizophrenia, under testing, not comercially available	[96]
SEN12333	5-morpholin-4-yl-pentanoic acid (4-pyridin-3-yl-phenyl)-amide		[95]
TC-5619	*N*-[(2*S*,3*S*)-2-(pyridin-3- ylmethyl)-1-azabicyclo[2.2.2]oct- 3-yl]-1-benzofuran-2- carboxamide		[102]
Clozapine	8-chloro-11-(4-methylpiperazin- 1-yl)-5Hdibenzo[ *b*,*e*][1,4]diazepine	Commercially available drug (trade names Azaleptin, Clozaril, FazaClo, Leonex and others), used as antipsychotics in paranoid disorders and schizophrenia	[51,53]
CNI-1493	*N*,*N*′-bis[3,5-bis[*N*- (diaminomethylideneamino)-Cmethylcarbonimidoyl] phenyl] decanediamide tetrahydrochloride	Inhibitor of inflammation and NO production, antagonist of α7 nAChR via cholinergic anti-inflammatory pahtway, known as a drug Semapimod, under clinical trials	[112–115]
GTS-21 (or DMXB-A)	3-[(3*E*)-3-[(2,4- dimethoxyphenyl)methylidene]-5,6-dihydro-4H-pyridin-2-yl] pyridine	Treatment of Alzheimer’s disease and cognitive deficits associated with schizophrenia, experimental testing for anti-inflammatory potency, under clinical testing	[107–110]
